# Trauma exposure and IPV experienced by Afghan women: Analysis of the baseline of a randomised controlled trial

**DOI:** 10.1371/journal.pone.0201974

**Published:** 2018-10-10

**Authors:** Rachel Jewkes, Julienne Corboz, Andrew Gibbs

**Affiliations:** 1 Gender & Health Research Unit, South African Medical Research Council, Pretoria, South Africa; 2 Independent Consultant, Kabul, Afghanistan; Stellenbosch University, SOUTH AFRICA

## Abstract

**Background:**

Four decades of conflict has indelibly impacted the lives of Afghans, exposing many to different forms of trauma. The aim of this paper investigate a hypothesis that (mostly war related) trauma is a key driver of partner violence in Afghanistan.

**Methods:**

1,463 women aged 18–48 were recruited into a randomised controlled trial (RCT) to evaluate a women empowerment intervention in 8 villages of Kabul and Nangarhar provinces. The women were interviewed at baseline. The analysis uses multivariable logistic regression and structural equation modelling (SEM) to describe relationships between measures.

**Results:**

57.4% of women reported exposure to one of four types of trauma: 23.3% an armed attack, 39.4% had felt close to death, 10.6% witnessed a friend or family member being killed and 21.4% witnessed the death of a stranger or someone unknown. Trauma exposure was associated with being older, Pashtan, madrassa educated, and food insecure. Women who were trauma exposed were more likely to have ever experienced IPV, have hit their children in the last 4 weeks, and be hit by a sibling or relative of their husband or their mother-in-law in the last year. They held less patriarchal personal gender attitudes and perceived the community to be more patriarchal. The SEM showed that all pathways between trauma exposure and IPV were ultimately mediated by either (mostly mental) ill-health or quarrelling, but not both of these. There were multiple paths through which trauma exposure impacted women’s past year experience of physical IPV. One was mediated by childhood trauma exposure and a latent variable for ill health. Other paths were mediated by women’s education and personal gender attitudes and ill-health, or else by quarrelling. Trauma exposure was related to lower educational levels. Another path was mediated by less patriarchal personal gender attitudes and ill health. Community gender attitudes was a mediating variable on a path which was also mediated by ill health and another mediated by quarrelling. It was also a mediator on a path which included personal gender attitudes and ill-health. Food insecurity mediated another path with ill health. It was also connected to childhood trauma, community gender attitudes and educational level.

**Conclusion:**

Trauma exposure due to conflict will persist until the conflict ends but the impact on women can be ameliorated. This analysis suggests interventions to reduce women’s exposure to IPV should focus on reducing poverty, changing social norms on gender, providing relationship skills to help reduce quarrelling and supporting women’s mental health.

## Introduction

War has a vast and enduring impact on the lives of affected civilian populations and in recent years there has been an increasing recognition that war also exacerbates violence against women and girls. Much attention has been given to rape in conflict, but there is a growing awareness that intimate partner violence (IPV) and violence against children are also increased during conflict and in post-conflict periods [1–5]. Further, war-related rape is not necessarily a predominant feature of all conflicts settings [6]. Research from several countries shows that both men and women’s experience of trauma in war is associated for women with a greater risk of experiencing IPV, and for men the risk of perpetration [1, 7].

Afghanistan is an important country for research on these issues as it has experienced four decades of conflict, which has impacted the entire country. It is also known to have a particularly patriarchal culture, especially in rural villages, which is associated in most settings with very high levels of violence against women. A Demographic and Health Survey was conducted in 2015 and it showed that the proportion of ever married women who have experienced physical, sexual or emotional violence from their husband in their lifetime ranges from over 90% in Herat and Ghor Provinces, to 7% in Badakhshan [8]. This shows that there is huge variation in social norms and practices within this conflict torn country. There has been little research conducted on the drivers of intimate partner violence against women in Afghanistan and the role of the conflict, if any, has not been explored.

In other settings, there has been much work on the impact of war trauma on (mostly male) combatants, and it is known that post-traumatic stress disorder and depression are increased, and that these are associated with a greater risk of men perpetrating violence against an intimate partner [9, 10]. Research has also shown that women who experience mental distress and have been trauma exposed are at greater risk of becoming victims of partner violence [11]. There are also a range of consequences of conflict which influence IPV, which in Afghanistan constitutes violence from a husband, including deepening poverty, disrupted families, reduced functioning of police and justice systems, growing acceptability of use of violence (as shown by increased use of many different forms of violence), and the undermining of traditional masculinities which may be pertinent in the Afghan setting [12–14]. In particular, conflict can reduce men’s opportunities for work, farming and livelihoods and thus reduce their ability to provide for their family, which may undermine their self-assessment of their performance as providers and protectors, and thus cause stress that can be expressed in use of violence [12, 14].

The baseline dataset for the evaluation of a women’s empowerment intervention in Afghanistan provided the opportunity to investigate the impact of trauma exposure on women’s views on gender and experiences of violence. The objectives of the paper are to describe the scope of trauma exposure from available variables, describe women’s social and demographic indicators associated with trauma exposure, measure the associations between trauma exposure and gender attitudes, women’s experience of violence and violence women use against their children, and use structural equation modelling to describe pathways from trauma exposure to past year physical IPV experience. The SEM model was theoretically built about well-established risk factors impacting women’s IPV risk [15]. However our overarching hypothesis was that woman’s trauma exposure in Afghanistan, predominantly due to war, would impact multiple risk factors. It would heighten stress in the home and result in increased exposure of children to trauma in the family. The trauma of war would negatively affect mental health, poverty, education and gender attitudes and due to well established pathways to physical IPV this would impact women’s risk of IPV [11, 16, 17]. For this analysis trauma exposure was defined as whether the woman had in her lifetime ever witnessed a family member or friend being killed, the death of a stranger or someone she knew, and a rocket attack or bomb falling or an armed attack on someone, and whether she had ever been or felt she was close to death.

## Methods

The interviews were conducted as the baseline for a randomised controlled trial (RCT) evaluation of the Women For Women International (WFWI) women’s economic and social empowerment intervention. This was part of the What Works To Prevent Violence? A Global Programme on Violence Against Women and Girls (VAWG), which has been funded by the UK Government’s Department For International Development (DFID) to advance the global knowledge on prevention of VAWG. The Global Programme is supporting the evaluation of 11 interventions using rigorous methods enabling an assessment of their effectiveness in preventing violence against women and girls. This evaluation is part of this overall portfolio of work.

We interviewed 1463 women who lived in Nangarhar and Kabul provinces of Afghanistan. They were recruited from six villages. The sample was thus a volunteer sample of women interested in participation in the intervention, who were then randomised into the two study arms. The eligibility criteria for the trial were being age 18–45 years, poor (preferably earning less that US$1 per day), able to consent and willing to participate in an intervention held over a year. The villages were selected by WFWI after taking into account a range of geographical, political and social considerations such that the intervention was predicted to be able to be feasibly and safely delivered in these settings. Women were randomised after recruitment to the intervention or control arm. Control arm women were given US$10 to incentivise the research interview. The number of women interviewed was determined by the sample size calculation for the main RCT.

Interviews were conducted face to face in a private community location (community centre or building serving this purpose) with a female interviewer. These were women from the provinces in which data was collected. All had completed secondary school and most had a background in teaching. Two were university students at the time of data collection. Most of them had several years’ experience in data collection. A standard questionnaire was developed and translated into Dari and Pashto (with back translation). It was pre-tested with 100 interviews and adjusted after the pre-test. Interviews were conducted with the language of choice for women and responses recorded on paper questionnaires. The questionnaires included questions on social and demographic characteristics of the women, their gender attitudes, perceived community gender attitudes, mental health, sexual and reproductive health and women’s exposure to violence. Most of the measures had established validity in other settings. After pre-testing we considered the responses to items and calculated Cronbach’s alphas to examine internal consistency of scales. We reduced the number of trauma exposures by two, as none of the women reported experiencing these (experience of being kidnapped, robbed at gun point), we deleted an item on whether women had ever attempted suicide, we simplified the contraception questions, and removed two items about the ‘honesty of reporting’ by participants.

Ethical approval for the research was given by the Ethics Committee of the Medical Research Council of South Africa and the Institutional Review Board of the Ministry of Public Health of Afghanistan. As a first stage WFWI connected with the villages and gained support for running the intervention in them. WFWI staff explained that there was to be random selection into the intervention and research conducted. These meetings were initially with community elders and men as well as women so that, as far as possible, women had family support for the research and, if selected, intervention participation. On the day of recruitment, all women interested in participation presented themselves to the site and were screened for trial inclusion. Those screened as eligible were allowed to enter the recruitment rooms where they consented for the study and were randomised into the intervention or control arm. Literacy levels were very low and so women were verbally informed about the research and given a written information sheet. They were asked to give consent on a form with a thumb print. Interviews were conducted on a subsequent day.

### Measures

Trauma exposure was measured by four questions asking whether the woman had in her lifetime ever witnessed a family member or friend being killed, the death of a stranger or someone she knew, and a rocket attack or bomb falling or an armed attack on someone, and whether she had ever been or felt she was close to death. Each item had a binary response. For the analyses presented, these responses were summed (used as a trauma exposure score in the SEM) and categorised into a two (ever/never for regression analyses) or three level (never, one, 2–4 exposures) variable. This item was developed for the study after discussion with Afghan WFWI staff about common exposures.

Physical violence from a husband was assessed with five questions which were taken from the World Health Organisation (WHO)’s instrument [18]. Each item asked about a specific act of violence and allowed for never, once, few or many responses. The questions were asked for ‘the last 12 months’ and lifetime. A measure of ever violence for each time frame was developed and used in logistic regression modelling, and for the SEM a score was used (generated by adding the items). The questions asked about acts such as whether the woman had been slapped, pushed, hit, threatened with a knife or gun, or had them used on her.

We measured current quarrelling with women’s husbands with one item that asked ‘in your relationship with your husband, how often would you say you quarrelled?’ and response options were rarely, sometimes, and often. This is a widely used item [15].

Violence from a mother in-law was asked of women who had met their mother in-law, even if she did not co-reside with them. They were asked if in the ‘last 12 months you were slapped, hit or beaten by your mother-in-law’. Responses were ‘never, sometimes, or often’. For logistic regression modelling a binary variable (ever/never) was derived.

Violence from another relative or in-law was asked of all women. They were asked if in the ‘last 12 months they were slapped, hit or beaten by their brother or sister or a brother or sister or other relative of your husband’. Responses were ‘never, sometimes, or often’. For logistic regression modelling a binary variable (ever/never) was derived.

The variable on hitting children was derived from a question asking “in the last 4 weeks, how often did you punish your children by giving a slap or beating or otherwise physically punishing them?”. It had response options for never, once, 2–3 times and 4 or more times and was used as a binary variable (ever/never) in logistic regression.

To measure gender attitudes we developed a series of questions that were specific for the Afghan context which is much more constrained for women than many contexts in which gender attitudes are often measured and so it was recognised that some of the commonly used measures (such as the GEM scale[19]) would not be appropriate. These were based on a question format that was initially developed in South Africa. Each question was asked twice, to separate thee personal views from perceived socially normative attitudes by starting each question with “my community thinks…” or “I think…”[20]. The actual content of the questions were developed with Afghan colleagues and perceived as relevant to the specific situation of Afghanistan. The scale items were then discussed with colleagues in Pakistan and refined. The scale was pre-tested among school children and two groups of adults in Afghanistan, and also among school children in Pakistan, before being used in this study. Each scale had 11 items and typical items were “I think the wives in my family should have a say in how money is spent” and “in this community most people think that girls should go to school”. Three items of each scale asked about attitudes towards wife beating. Cronbach’s alpha for the community scale was 0.90 and for the personal scale was 0.87.

Childhood trauma was measured on a 11 item scale adapted from Bernstein [21]. It was phrased “before I was married’ as in Afghanistan that is considered the period of childhood and women may marry before 18, and in some cases afterwards. Two items measured not being taken care of, three physical neglect or other hardship (not having enough to eat, living in different households at different times and having to work), emotional abuse (including two items on witnessing her mother being beaten, and two on being verbally abused) and physical abuse (two items). We were advised that asking about sexual abuse would be very sensitive and so did not include such questions. The measure was operationalised as a score for the SEM by summing the responses.

We measured depression using the 20 item CES-D scale [22]. We have used it as a continuous variable in the SEM model in part because it has not been validated for use in women in Afghanistan and the appropriate cut point for a highly patriarchal and conflict setting is not known.

We measured PTSD symptoms using the Harvard Trauma Questionnaire [23]. In the SEM we used a measure of PTSD symptom mean which was the sum of the 16 items divided by 16. Again this was done because we did not have the requisite information for PTSD diagnosis (i.e. symptom duration, trauma exposure and frequency). We measured disability using the six item Washington Group scale [24] and summed these as the disability measure for use in the SEM. A typical items was “Do you have difficulty walking or climbing steps?”

In addition, we asked about age, highest level of schooling (none, madrassa, primary or secondary) and marital status. We assessed poverty by asking three questions about frequency of food insecurity in the last 4 weeks, whether: there had being no food to eat in the house due to lack of money, a member of the household went to sleep without eating due to lack of food, and a member of the household went all day and night without eating due to lack of food. Responses were never, rarely, sometimes and often. These were summed (range 3–12) and a three level variable derived with no (answering ‘no’ to all questions), moderate (score 4–6) and severe food insecurity (responding ‘often’ to one item or scoring overall 7 or more).

### Data analysis

Questionnaires were doubled entered into SPSS databases that were merged and verified. Data was analysed in Stata 13.0. Missing data was <5% (for example 3/935 women lack past 12 month IPV data) and imputation was not used. Continuous variables were summarised using means and 95% confidence intervals and categorical variables with percentages and chi squared tests. We used multivariable logistic regression to model social and demographic factors associated with trauma exposure and selected variables based on an a priori decision about the more appropriate measures. In order to examine associations between trauma exposure and personal and community gender attitudes, women’s husband and in-law violence experience and use of violence against her children, we built a multivariable logistic regression model for each categorical outcome and multivariable regression model for continuous outcomes and modelled independent variables which were the social and demographic characteristics and war exposure. We present the full models without elimination of variables. The model chi squared is presented for each, as is the sample size. Measures are presented for the whole sample, or for just married women (smaller sample sizes).

Structural Equation Modeling (SEM) was conducted using Stata 15.0 to assess the interrelationship between variables associated with lifetime physical IPV. The model outcome was a past year physical IPV score based on responses to five questions about whether a physical IPV act had been experienced not at all, once, or few or many occasions in the past year. The correlation between each hypothesized variable and the IPV variable was then tested by building variable pairs. All associations were tested by running a full-information maximum likelihood method to deal with missing values. This method was chosen over multiple imputations as it has been shown to yield superior results in structural equation modeling [25]. As a next stage, a measurement model was fitted with the variables allowed to freely correlate. To assess model fit of the observed data, we used the comparative fit index (CFI) (>0.95); Tucker-Lewis Index (TLI) (>0.9) for acceptable fit and (>0.95) as indicative of good fit[26]; and root mean square error of approximation (RMSEA) (of 0.05 or less)[27, 28].

We fitted a path model using full information maximum likelihood estimation (FIML) to model all available data. The final model was built based on theory and statistically meaningful modifications using backwards elimination to exclude endogenous variables that did not mediate any path (with significance set at the p < .05 level) from the exogenous variables to past year IPV in order to ensure model parsimony. We co-varied errors to improve model fit where we could justify this theoretically. This resulted in the following covarying errors: childhood trauma and personal gender attitudes, food insecurity and ill-health, food insecurity and depression, PTSD and disability, depression and disability, personal gender attitudes and disability, and education and health.

## Results

The trauma exposure of the women interviewed is shown in Table 1. In response to the four questions, 57.4% of women reported trauma exposure: 32.4% had one exposure and 16.8% had two, 4.8% had three and 3.5% had all four of the trauma exposures. Most commonly women reported that they had been or felt they were close to death (39.4%) but one in five had witnessed the death of a stranger or someone they knew and a similar proportion had witnessed a rocket attack or bomb falling or an armed attack on someone. Less commonly they had witnessed the death of a family member or friend (10.6%).

**Table 1:** Trauma exposure among a sample of Afghan women.

Trauma exposure and other trauma	Yes		No		
	%	n	%	n	N
Witnessed a family member or friend being killed	10.6	155	89.4	1306	1461
Witnessed the death of a stranger or someone you knew	21.4	313	78.6	1148	1461
Witnessed a rocket attack or bomb falling or an armed attack on someone	23.3	339	76.7	1119	1458
Has been or felt you were close to death	39.4	570	60.6	878	1448
Trauma exposure score: none	42.6	622			1416
1	32.4	473			
2	16.8	245			
3	4.8	70			
4	3.5	51			
Any trauma exposure	57.4	839	42.6	622	1461
trauma exposure: none	42.6	622			1461
1	32.4	473			
2–4 exposures	25.1	366			

The mean age of the women was 29.28 (range 14–48). 59.8% of women were currently married and 6.3% previously married. Table 2 shows that there was a dose response relationship between trauma exposure and age so that with the mean age of each trauma exposure category was significantly higher than the previous one. For ethnicity, 53.5% were Tajik, 25.2% were Pashtun and 19.8% were Hazara, and 1.6% had other ethnicities. Trauma exposure was significantly associated with ethnic group, with only 22.2% of Pashtuns having none compared to 47.3% of Tajik women and 56.4% of Hazara women. However the proportion in the highest trauma exposure category was quite similar for Pashtun (26.3%) and Tajik (29.4%) women. Overall 77.1% of women had no education, 10% had primary schooling, 6% had secondary and 6% were educated in a madrassa (religious education only). Trauma exposure was lower among more educated women. In terms of food security, 60% were food secure, 33% had moderate insecurity and for 6.9% this was severe. There was significantly more food insecurity among trauma exposed women. Never married women had less trauma exposure than those currently or previously married.

**Table 2:** Social and demographic characteristics, gender attitudes, health and violence exposure and war exposure.

	Trauma exposure							
	None		1		2–4			
	%	n	%	n	%	n	N	p value
**Mean age**	26.74	26.11, 27.37	29.6	28.83, 30.37	33.23	32.22, 34.13		<0.0001
**Ethnic group:** Pashtun	13.18	82	40.2	190	26.5	97	369	<0.0001
Tajik	59.32	369	38.5	182	62.6	229	780	
Hazara	26.2	163	19.2	91	9.6	35	289	
Other	1.29	8	2.1	10	1.4	5	23	
**Education group:** None	72.3	447	78.8	372	83.0	303	1122	<0.0001
Madrassa	4.2	26	8.7	41	7.1	26	93	
Primary	14.2	88	7.6	36	6.3	23	147	
Secondary	9.2	57	4.9	23	3.6	13	93	
**Food insecurity:** None	70.1	435	53.7	254	51.4	188		<0.0001
Moderate	26.3	163	37.8	179	38.3	140		
Severe	3.7	23	8.5	40	10.4	38		
**Marital status:** Married	57.6	358	67.4	319	70	256	933	<0.0001
Previously married	4.2	26	6.3	30	11.5	42	98	
Never married	38.3	238	26.2	124	18.6	68	430	
**Gender attitudes (personal)**	22.48	22.24, 22.71	21.76	21.47, 22.05	22.19	21.83, 22.55	1460	0.002
**Perception of community gender attitudes**	21.45	21.22, 21.67	22.35	22.03, 22.66	22.94	22.55, 23.34	1460	<0.0001
**Past 12 months physical IPV**	17.7	63	27.6	88	25.1	64	931	0.006
**Lifetime physical IPV**	28.1	103	47.0	155	44.6	124	974	<0.0001
**Hit child in last 4 weeks**	46.3	142	55.9	189	56.9	152	895	0.003
**Beaten by sibling or in-law in last 12 months**	9.8	60	16.9	79	14.9	54	1441	0.002
**Beaten by mother-in-law in last 12 months**	8.4	20	15.4	35	16.6	26	624	0.025
**Depression**	12.05	11.40, 12.70	16.25	15.34, 17.16	17.1	15.94, 18.25	1459	<0.0001
**PTSD**	1.35	1.31, 1.38	1.7	1.66, 1.74	1.65	1.59, 1.71	1461	<0.0001
**Disability**	30.2	148	36.9	181	44.4	161	1444	<0.0001

Personal gender attitudes were significantly less patriarchal among women who had a trauma exposure, but they perceived the gender attitudes of their community to be more patriarchal (Table 2). There was a significantly higher proportion of women reporting past year (17.7% v. 27.6%) and lifetime (28.1% v. 47.0%) exposure to IPV comparing those with no trauma exposure and those with one exposure, with the same pattern seen for the higher exposure group. Women with trauma exposure once were more likely to have beaten a child in the last 4 weeks than those without (46.3% v. 55.9%) and were much more likely to have been beaten by their mother-in-law or a sibling or other in-law. They were significantly more likely to have more depressive symptoms, symptoms of PTSD and there was a dose response relationship between trauma exposure and disability, such that the proportion of women with disability increased with increasing trauma exposure.

Trauma exposure was associated with increasing age, was less common among Tajiks and Hazaras than Pashtuns, was more commonly reported by women educated in madrassa’s than those never educated, and was associated with greater food insecurity (Table 3).

**Table 3:** Socio-demographic factors associated with trauma exposure.

	Any trauma exposure			
	aOR	95% CI		P value
Age	1.08	1.06	1.09	<0.0001
**Ethnicity:** Pashtun	ref			
Tajik	0.28	0.20	0.38	<0.0001
Hazara	0.20	0.14	0.29	<0.0001
Other	0.50	0.19	1.34	0.168
**Education:** None	ref			
Madrassa	2.22	1.34	3.68	0.002
Primary	0.92	0.63	1.37	0.692
Secondary	1.09	0.68	1.75	0.729
**Food insecurity:** None	ref			
Moderate	1.46	1.13	1.88	0.003
Severe	1.42	0.84	2.42	0.191

Table 4 shows the associations between trauma exposure and experience and use of violence by Afghan women. After adjusting for social and demographic factors, trauma exposure was significantly associated with life time IPV and there was a suggestion that it may have elevated the odds of past year IPV aOR 1.27 (95%CI 0.89, 1.83 p = 0.188), although this was not statistically significant. There was considerable evidence that it was associated with having been beaten by her mother in-law in the past year aOR 1.72 (95%CI 0.98, 3.04 p = 0.06). It was associated with having been beaten in the past year by a sibling or other relative of her husband aOR 1.50 (95%CI 1.04, 2.16), and with women having beaten their own children in the last four weeks aOR 1.40 (95%CI 1.04, 1.89). Trauma exposed women had significantly less patriarchal personal gender attitudes, but perceived their community’s gender attitudes to be more patriarchal.

**Table 4:** Associations between trauma exposure, gender attitudes, and violence experience and use by Afghan women.

	**Lifetime physical IPV**				**Hit child in last 4 weeks**				**Hit by a sibling or relative of your husband in last 12 months**				**Hit by your mother-in-law in last 12 months**			
	**aOR**	**95% CI**	** **	**P value**	**aOR**	**95% CI**	** **	**P value**	**aOR**	**95% CI**	** **	**P value**	**aOR**	**95% CI**	** **	**P value**
**Trauma exposure**	1.69	1.24	2.31	0.001	1.40	1.04	1.89	0.028	1.50	1.04	2.16	0.029	1.72	0.98	3.04	0.06
**Mean age**	1.03	1.01	1.06	0.001	1.00	0.98	1.02	0.971	0.96	0.94	0.98	<0.0001	1.01	0.97	1.05	0.547
**Ethnic group:** Pashtun																
Tajik	0.56	0.39	0.80	0.002	0.54	0.37	0.77	0.001	0.35	0.24	0.52	<0.0001	0.58	0.32	1.05	0.072
Hazara	1.05	0.69	1.61	0.816	0.78	0.50	1.21	0.271	0.66	0.41	1.05	0.082	1.28	0.66	2.47	0.47
Other	1.94	0.61	6.16	0.263	2.17	0.58	8.04	0.247	0.42	0.12	1.51	0.185	0.27	0.03	2.28	0.23
**Education group:** None																
Madrassa	2.23	1.19	4.19	0.013	0.87	0.45	1.65	0.661	1.01	0.57	1.81	0.964	2.26	0.90	5.67	0.082
Primary	1.75	1.02	2.98	0.041	1.12	0.62	2.00	0.713	0.84	0.48	1.49	0.556	0.99	0.41	2.40	0.984
Secondary	0.56	0.21	1.51	0.248	0.67	0.28	1.58	0.359	0.60	0.26	1.37	0.224	1.46	0.39	5.44	0.569
**Food insecurity:** None																
Moderate	2.95	2.19	3.97	<0.0001	2.70	1.99	3.68	<0.0001	1.85	1.31	2.63	0.001	2.10	1.24	3.56	0.006
Severe	2.87	1.62	5.11	<0.0001	1.27	0.73	2.21	0.404	2.70	1.56	4.67	<0.0001	2.89	1.18	7.06	0.02
	(n = 960 women, p = 0.0001)				(n = 892, p = 0.0001)			(n = 1434, p<0.0001)					(n = 620, p = 0.0007)			
	**Patriarchal gender attitudes**				**Patriarchal gender attitudes perceived in community**				**Past 12 months exposure to physical IPV**							
	**Coeff**	**95% CI**	** **	**P value**	**Coeff**	**95% CI**	** **	**P value**	**aOR**	**95% CI**	** **	**P value**				
**Trauma exposure**	-0.85	-1.20	-0.50	<0.0001	0.45	0.10	0.79	0.011	1.27	0.89	1.83	0.188				
**Mean age**	0.00	-0.02	0.02	0.82	0.00	-0.02	0.02	0.771	1.02	0.99	1.04	0.179				
**Ethnic group:** Pashtun																
Tajik	-0.45	-0.87	-0.03	0.037	-1.97	-2.38	-1.56	<0.0001	0.46	0.31	0.69	<0.0001				
Hazara	-1.64	-2.16	-1.12	<0.0001	-1.88	-2.39	-1.37	<0.0001	0.76	0.47	1.20	0.238				
Other	-2.33	-3.68	-0.98	0.001	2.98	1.67	4.30	<0.0001	0.37	0.09	1.44	0.151				
**Education group:** None															
Madrassa	-1.23	-1.90	-0.55	<0.0001	0.15	-0.51	0.81	0.662	1.47	0.74	2.92	0.268				
Primary	-0.04	-0.61	0.52	0.882	0.57	0.02	1.12	0.044	0.91	0.47	1.74	0.773				
Secondary	-1.10	-1.79	-0.40	0.002	-0.47	-1.14	0.21	0.176	0.77	0.26	2.31	0.64				
**Food insecurity:** None																
Moderate	0.58	0.23	0.94	0.001	1.48	1.13	1.83	<0.0001	2.77	1.97	3.89	<0.0001				
Severe	0.40	-0.28	1.09	0.248	1.70	1.03	2.37	<0.0001	3.46	1.84	6.51	<0.0001				
	(n = 1453, p<0.0001)				(n = 1453, p<0.0001)				(n = 927, p<0.0001)							

**Fig 1:**
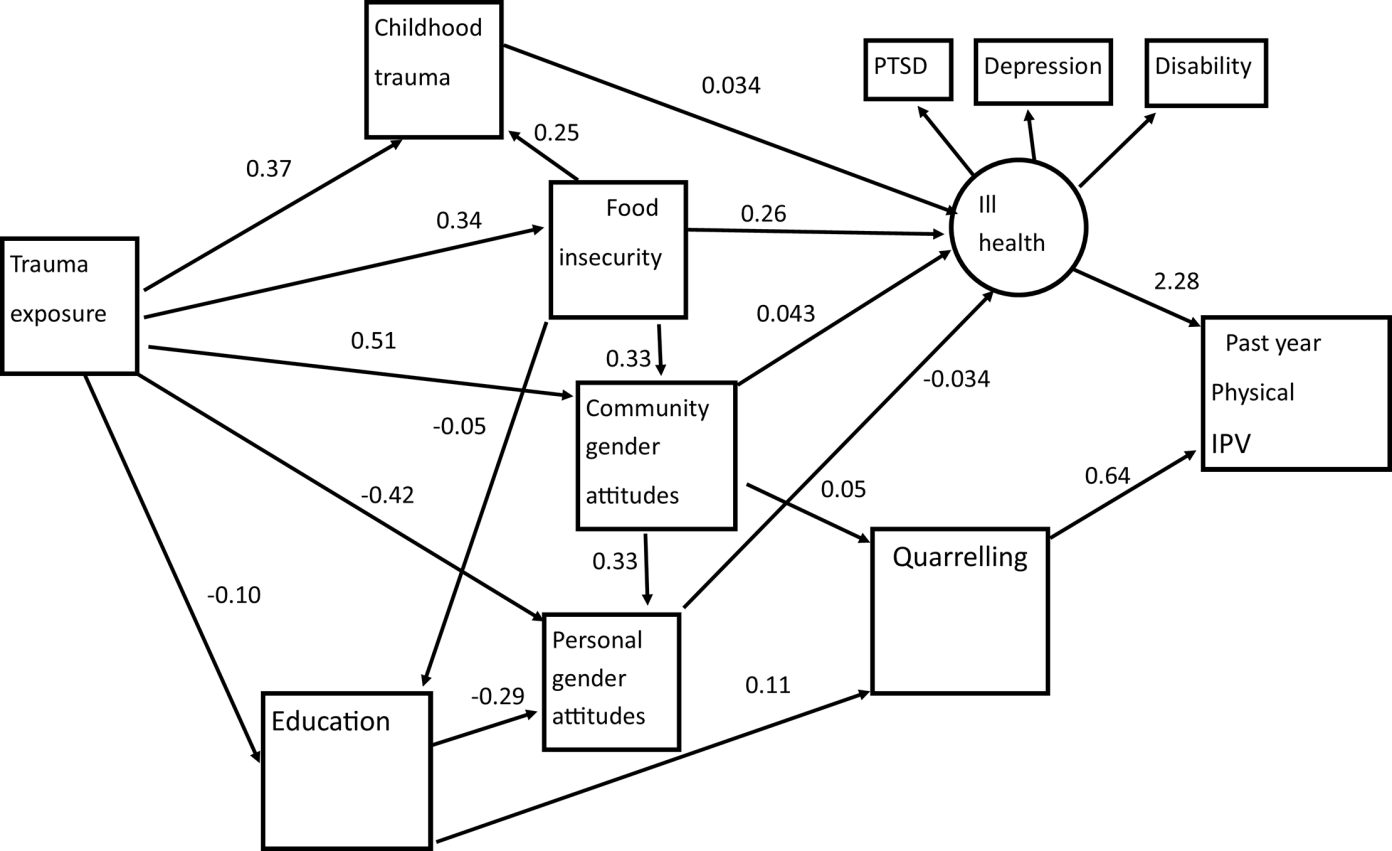
Structural model of factors influencing women's experience of physical intimate partner violence (IPV) in the past year (standardized path coefficients {only statistically significant paths shown}).

The SEM shows two final common pathways to IPV experience which were mediated by ill-health and quarrelling but not by both (Fig 1. and Table 5). All the pathways were mediated by a combination of personal and community gender attitudes, women’s education, food insecurity and childhood trauma. The direction of effect shows that women with less patriarchal personal attitudes had elevated IPV experience, with this mediated by poorer (mental) health. Conversely women who perceived their community’s attitudes to be more patriarchal also had poorer health and there was more quarrelling and elevated IPV experience. Women’s education was associated with having less patriarchal personal gender attitudes and so is seen to elevate IPV experience. Childhood trauma and food insecurity elevated ill health.

**Table 5:** Path model: Direct effects, disturbance variances and equation-level goodness of fit.

Parameter	Standardized coefficients	SE	z	P>|z|	[95% Conf. Interval]	
Direct effects						
childhood trauma—> trauma exposure	0.3660	0.0822	4.45	<0.0001	0.2048	0.5272
trauma exposure—> community gender attitudes	0.5132	0.0799	6.42	<0.0001	0.3565	0.6699
trauma exposure—> personal gender attitudes	-0.4179	0.0762	-5.48	<0.0001	-0.5774	-0.2685
trauma exposure—> food insecurity	0.3433	0.0506	6.78	<0.0001	0.2441	0.4424
trauma exposure—> education	-0.0978	0.0226	-4.34	<0.0001	-0.142	-0.0535
Food insecurity—> education	-0.0459	0.0082	-5.58	<0.0001	-0.0621	-0.0298
Food insecurity—> childhood trauma	0.2501	0.0298	8.38	<0.0001	0.1915	0.3086
Food insecurity—> community gender attitudes	0.3303	0.0291	11.34	<0.0001	0.2733	0.3874
Food insecurity—> ill health	0.2639	0.0291	9.08	<0.0001	0.2069	0.3208
Community gender attitudes—> personal gender attitudes	0.3292	0.0234	14.08	<0.0001	0.2834	0.3751
Community gender attitudes—> quarrelling	0.0502	0.0092	5.47	<0.0001	0.0322	0.0682
Community gender attitudes—> illhealth	0.0429	0.0033	12.82	<0.0001	0.0363	0.0494
Personal gender attitudes—> illhealth	-0.0338	0.0036	-9.50	<0.0001	-0.0408	-0.0268
Childhood trauma—> ill health	0.0337	0.0033	10.30	<0.0001	0.0273	0.0401
Education—> personal gender attitudes	-0.2898	0.08598	-3.38	0.001	-0.4579	-0.1217
Education—> quarrelling	0.1052	0.0392	2.68	0.007	0.0283	0.1821
Quarrelling—> IPV	0. 6373	0.07576	8.41	<0.0001	0.4879	0.7858
illhealth—> IPV	2.2755	0.1636	13.91	<0.0001	1.9550	2.5961
Equation-level goodness of fit	r-squared					
PTSD	0.7891					
Depression	0.7431					
Disability	0.4176					
IPV	0.2734					
Community gender attitudes	0.1157					
Personal gender attitudes	0.1274					
Education level	0.0375					
Quarrelling	0.0448					
Childhood trauma	0.0643					
Food insecurity	0.0157					
Ill health (latent)	-1.2123					
Overall	0.1485					

The ill-health latent variables was indicated by PTSD, depression and disability. There were multiple paths through which trauma exposure impacted women’s past year experience of physical IPV. One was mediated by childhood trauma exposure and a latent variable for ill health. Other paths were mediated by women’s education and personal gender attitudes and ill-health, or else by quarrelling. Trauma exposure was related to lower educational levels. Another path was mediated by less patriarchal personal gender attitudes and ill health. Community gender attitudes was a mediating variable on a path which was also mediated by ill health and another mediated by quarrelling. It was also a mediator on a path which included personal gender attitudes and ill-health. Food insecurity mediated another path with ill health. It was also connected to childhood trauma, community gender attitudes and educational level. The model fit was good Chi squared p<0.0001, RMSEA 0.043, CFI 0.981 and TLI 0.962.

## Discussion

Conflict has been a central feature of life in Afghanistan for the greater part of the last 4 decades. Understanding the impact of trauma on Afghan women’s lives is very important. A high proportion of women in Afghanistan interviewed for this study had experienced one or more traumatic events in their lifetime, which for all spanned decades of conflict. We have shown that beyond the immediate experience, trauma exposure, much of which was conflict-related, impacted their level of household wealth, as indicated by food insecurity. Although we do not know if current poverty reflects childhood poverty, it seems likely that they are related and that the patterns we describe are those of a vicious circle whereby the heightened poverty enhanced the vulnerability of the women’s childhood, which in turn influenced their vulnerability to trauma. Trauma also impacted on levels of education, emphasising that exposure to traumatic events are a proxy for the wider exclusion of women and girls from education, either through the destruction of schools, or through girls being kept inside homes to try and protect them from the conflict. We have shown that trauma exposure impacted women’s perceptions of attitudes towards gender in the community, and trauma exposed women perceived communities to be more patriarchal, which impacted on the (mostly mental) health and overall experience of partner violence. It is well recognised that conflict situations often require women to take on roles normally considered domains of men. Whilst this may be muted on a country like Afghanistan which is so highly patriarchal, the need for women to be more independent may be perceived even if the realisation is somewhat frustrated. This may impact individual gender attitudes and community perceptions.

Many of the associations shown in the model have been well described in the literature on factors associated with intimate partner violence. The association of IPV with mental health is well recognised [11, 29, 30]. It is generally considered to be a two way association such that women’s poor mental health drives IPV and IPV impacts women’s mental health. In this model the major direction of impact was of (largely mental) ill health causing IPV experience. The latent variable included a measure for disability, which measured aspects of mental disabilities e.g. poor concentration. It is increasingly recognised that disability renders women more vulnerable to IPV, although of course IPV can also cause physical, mental and sensory disability [31, 32]

The association between quarrelling and violence is well recognised as violence is often used as a tactic in conflict [15, 33]. The structural model points to the impact of patriarchal community attitudes on quarrelling. It’s likely that the quarrelling is a product of perceived gender norms transgressions or restrictions. Similarly the association between food insecurity and childhood trauma is well described [16]. Research in Asia has previously demonstrated an association between greater poverty and social conservatism i.e. perceiving more conservative community attitudes and holding more patriarchal personal attitudes [15].

### Limitations

This analysis was based on data from a non-randomised sample, but there was no reason to imagine that the associations between variables to be influenced by this, so we would consider the associations found to more widely pertain in the area in which the women lived. Our IPV measure was limited to physical IPV as it is very sensitive discussing sexual violence in Afghanistan and we were worried that including questions might influence the overall acceptability of the study in the community. We recognise the 2015 Afghanistan DHS did ask about sexual violence but the very low levels (7.4% lifetime exposure) reported probably reflect this sensitivity and confirms our decision not to ask about sexual violence. There is alcohol and drug use by men in Afghanistan which might have impacted on IPV experience but alcohol use is illegal, not terribly common and we were unable to ask about it. Drug use, especially opiates, is more common but our question on husband’s drug use was misinterpreted by women as including tobacco and so we have not included it in analyses. We are aware that physical IPV may have been under-reported by women due to concerns about family privacy, but the lifetime experience at 39.2% was a little higher than that reported in the DHS for Kabul and lower than Nangarhar (37.9% for Kabul and 56.0% for Nangarhar) and so there may have been some under-reporting. We recognise that we only asked four questions about trauma exposure and women will have had many other exposures and aspects of their lives impacted by conflict, but we consider these four as very severe and indicative of broader patterns. This study was based on cross-sectional data and we are unable to have confidence that the trauma exposure temporally preceded the IPV experience. Since the proportions are very similar, the DHS showed that most Afghan women who had ever been beaten (50.8%) had been beaten by their husband or experienced sexual IPV had done so in the past year (46.1%). In our sample the difference between lifetime and past year exposures was much larger (39.2% and 23.1%) which may reflect under-reporting of recent IPV or the non-random nature of sample selection. Overall the R squared of the SEM is not very large, but this is not really surprising as we have been only able to assess women’s factors in this study and since IPV is a behaviour perpetrated by men it is inevitable that many of the factors would be unmeasured.

## Conclusions

Trauma exposure is very common for Afghan women and most of the women interviewed would have lived their whole life through conflict. It was most experienced by Pashtun women who predominantly came from Nangarhar Province which has been at the centre of fighting for four decades, although women from Kabul have also been widely exposed. It is impossible to end the impact of conflict before the conflict itself is ended, but it is valuable to identify some of the pathways where there can be intervention to ameliorate the impact of trauma. Interventions that could make the lives for women better would impact reducing poverty, changing community norms on gender, providing relationship skills and providing support and treatment for women with mental health problems. Interventions such as that provided by Women For Women International [34] have the potential to impact on IPV as they address many of these. Evaluating such interventions to ensure that we have a solid foundation from which to prevent violence against women and girls in chronically trauma torn areas such as Afghanistan is essential.
